# Beyond the Reported Numbers: *Clostridioides difficile* Dominance (CDI) and Surveillance Bias in Healthcare-Associated Infections in Post-Pandemic Southeast Romania

**DOI:** 10.3390/antibiotics15070662

**Published:** 2026-07-04

**Authors:** Alina Plesea Condratovici, Mihaela Debita, Valerian Ionut Stoian, Catalin Plesea Condratovici, Ancuta Elena Tupu, Simona Steliana Tudor

**Affiliations:** Faculty of Medicine and Pharmacy, Medical-Pharmaceutical Research Center, “Dunarea de Jos” University of Galati, 800008 Galati, Romania; alina.plesea@ugal.ro (A.P.C.); catalin.plesea@ugal.ro (C.P.C.); anca.tupu@ugal.ro (A.E.T.); steliana.tudor@ugal.ro (S.S.T.)

**Keywords:** healthcare-associated infections, *Clostridioides difficile*, surveillance, under-reporting, in-hospital mortality, competing risks, funnel plot, post-pandemic, Romania, patient safety

## Abstract

**Background/Objectives:** Healthcare-associated infections (HAIs) are a major and preventable threat to patient safety, yet reported figures in Central and Eastern Europe are widely affected by under-reporting, which can distort both the apparent infection profile and the perceived burden of disease. Patient-level regional surveillance data were analysed to characterise the reported HAI profile, the determinants of in-hospital mortality, and between-hospital surveillance quality in post-pandemic Southeast Romania. **Methods:** This was a retrospective, cross-sectional analysis of 2878 HAI cases reported across a five-county, multicentre network of 32 hospitals during 2024. Infections were grouped as *Clostridioides difficile* infection (CDI) versus non-CDI. Logistic regression was applied for in-hospital mortality, Cox and competing-risks models for time to death, negative binomial regression for length of stay, and a Spiegelhalter funnel plot for between-hospital variation. **Results:** *Clostridioides difficile* infection accounted for 56.3% of reported cases, a markedly higher proportion than that described in European point-prevalence surveys, although differences in design and denominator preclude direct comparison. CDIs and non-CDIs formed distinct clinical phenotypes. In-hospital mortality was lower in CDI than in non-CDIs (14.9% versus 26.1%) and was independently associated with intensive care admission, age, and immunosuppression, while CDI remained associated with lower mortality. The reported CDI proportion ranged from approximately 1% to 93% between hospitals, with most institutions lying outside the funnel control limits. **Conclusions:** The predominance of CDI among reported HAIs is best interpreted as a signal of selective ascertainment rather than as direct evidence of a genuinely higher CDI burden. Because the dataset lacked admission or patient-day denominators, the CDI-to-total ratio should be regarded as a simple screening indicator of potential surveillance imbalance, useful for identifying hospitals where non-CDIs may be under-detected.

## 1. Introduction

Healthcare-associated infections (HAIs) remain among the most important and preventable threats to patient safety worldwide, contributing substantially to morbidity, mortality, prolonged hospitalisation, antimicrobial consumption, and healthcare costs [1,2,3,4]. The World Health Organization has therefore placed infection prevention and control at the centre of the global patient-safety agenda, emphasising the need for reliable surveillance systems capable of measuring the true burden of avoidable harm [1,5]. Despite progress in prevention, HAIs continue to represent a persistent and only partially controlled challenge across health systems of all income levels [2,6].

In Europe, harmonised point-prevalence surveys have shown that respiratory tract infections, urinary tract infections, surgical site infections, and bloodstream infections account for a large share of HAIs, with many cases involving multidrug-resistant organisms [7,8,9]. However, reported HAI rates vary markedly between countries, regions, and institutions. This variation reflects not only genuine epidemiological differences, but also differences in diagnostic capacity, reporting culture, case ascertainment, and the maturity of surveillance systems [10,11]. The distinction is particularly important in Central and Eastern Europe, where underdiagnosis and underreporting have repeatedly been identified as major limitations, and where apparently low HAI rates may indicate incomplete surveillance rather than a genuinely lower burden of infection [7,8].

The COVID-19 pandemic further complicated this landscape. Overcrowded hospitals, pressure on intensive care units, redistribution of staff and resources, increased use of invasive devices, and disruption of routine infection-prevention activities were associated with increased infection risk and changes in HAI epidemiology [12,13,14,15]. At the same time, the pandemic accelerated investment in microbiological diagnostics, reinforced infection-control structures, and increased awareness of patient safety and surveillance [16,17]. Whether these competing effects produced durable improvements in HAI detection and reporting after the acute pandemic phase remains uncertain, particularly in settings where surveillance systems were already affected by under-reporting [18,19].

**Figure 1:**
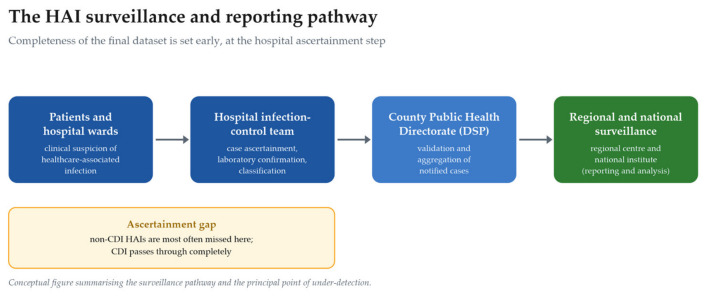
The HAI surveillance and reporting pathway, with the ascertainment gap.

In Romania, officially reported HAI rates have historically been lower than those observed in many European countries, a pattern widely interpreted as reflecting under-diagnosis and under-reporting rather than a genuinely smaller burden of infection [20,21]. Successive legislative, organisational, and methodological reforms have attempted to strengthen surveillance, clarify the responsibilities of infection-prevention structures, and standardise reporting practices, with the pandemic accelerating several of these processes [20]. Nevertheless, the completeness of *C. difficile* surveillance itself varies widely across Europe, with pronounced between-country and between-hospital differences in testing and reporting practice that produce large discrepancies in apparent CDI rates [22,23,24]. Differences in hospital infrastructure, microbiological capacity, specialised personnel, and local reporting culture are therefore likely to translate into substantial variation in surveillance performance between regions and between individual hospitals, from the bedside to national reporting (Figure 1) [18,25].

**Figure 2:**
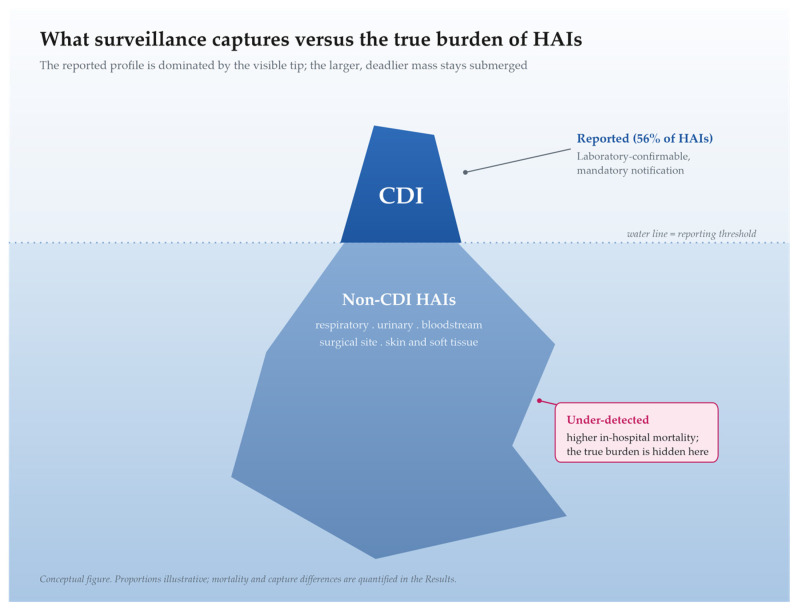
The HAI surveillance iceberg: the reported tip (CDI) versus the under-detected true burden (non-CDI).

An important but underappreciated feature of HAI surveillance in this context is the disproportionate contribution of *Clostridioides difficile* infection (CDI). Because CDI is laboratory-confirmable and subject to mandatory notification, it is more likely to be detected and reported than many other HAIs, which often depend on complex clinical assessment, microbiological confirmation, active case finding, and clinician-initiated reporting [26,27]. As a result, a surveillance system may appear to be dominated by CDI, not because CDI represents the true majority of HAIs, but because other infection categories are less completely captured. This imbalance can distort the apparent infection profile and may obscure the true mortality burden associated with under-reported non-CDIs (Figure 2).

Most Romanian studies of HAIs have been limited to single centres, specific infection categories, or short observation periods, and patient-level multicentre evidence from the post-pandemic period remains scarce [20,21]. In particular, the relationship between the reported infection profile, patient outcomes, and between-hospital variation in surveillance performance has not been well characterised. To address this gap, we analysed individual patient-level HAI surveillance data from a multicentre regional network in Southeast Romania during the complete 2024 reporting year.

The objectives of this study were threefold: first, to describe the epidemiological profile of reported HAIs and quantify the extent of CDI dominance; second, to compare the clinical characteristics and outcomes of CDI and non-CDIs and identify independent determinants of in-hospital mortality; and third, to assess between-hospital heterogeneity in the reported CDI proportion as a potential indicator of surveillance quality. By linking the reported infection profile to patient outcomes and institutional reporting patterns, this study aims to clarify what HAI surveillance captures in post-pandemic Romania and to inform the transition toward more complete, standardised, and data-driven surveillance systems [28,29].

## 2. Results

### 2.1. Cohort Characteristics

**Figure 3:**
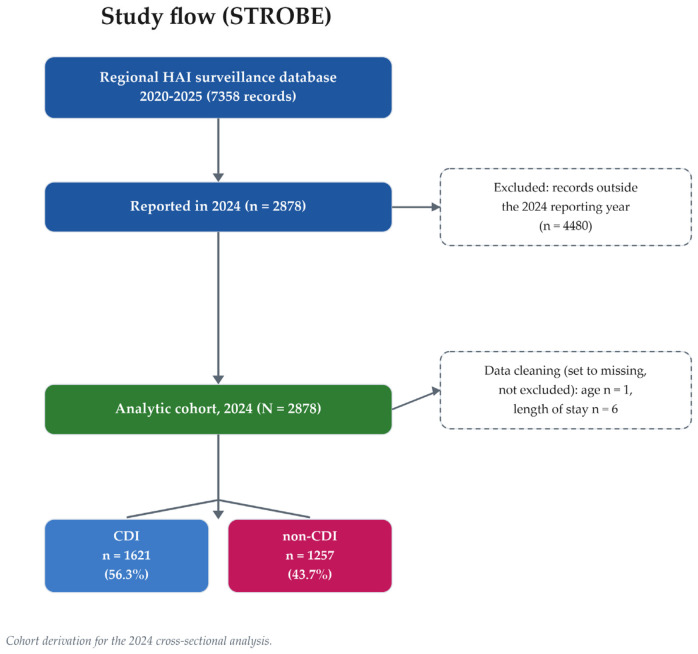
Study flow: derivation of the 2024 analytic cohort (N = 2878).

During the 2024 reporting year, 2878 healthcare-associated infection cases from the regional network entered the analytic cohort (Figure 3).

The median age was 68 years (IQR 52–76), the mean age was 60.6 years (SD 23.7), and 58.1% of patients were aged 65 years or older. Men accounted for 52.9% of cases with sex recorded, and 57.6% of patients resided in urban areas. Immunosuppression was documented in 23.6% of cases and hospitalisation within the preceding year in 28.2%; mechanical ventilation was rare (0.5%). Recent antisecretory therapy was recorded as a CDI-specific surveillance variable and was documented in 47.6% of CDI cases; it was therefore not included in the comparative baseline table or used for between-group inference. The median length of stay was 13 days (IQR 7–22). In-hospital mortality, assessed among the 2316 patients with a recorded discharge status, was 19.5% (451 deaths). The full baseline profile, overall and by infection group, is presented in Table 1.

### 2.2. Distribution of Infection Types and CDI Dominance

**Figure 4:**
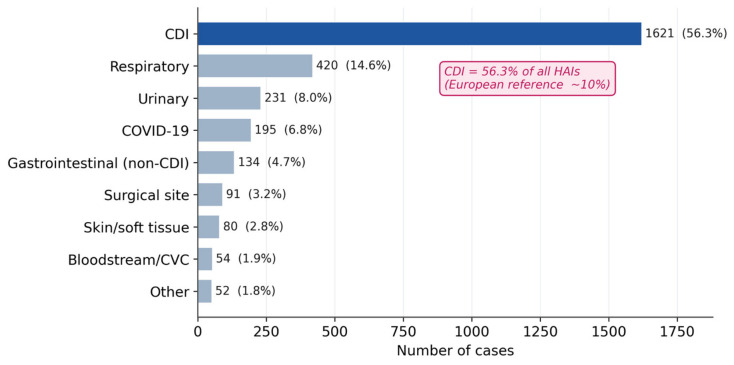
Distribution of healthcare-associated infection types, 2024 (N = 2878).

The reported HAI profile was dominated by a single entity. *Clostridioides difficile* infection accounted for 1621 of 2878 cases (56.3%), exceeding all other infection categories by a wide margin. This proportion is substantially higher than the approximately 10% reported for CDI in European point-prevalence surveys, although the difference should be interpreted cautiously because the present study used notified case-based surveillance data rather than a point-prevalence design with a defined hospital denominator. The remaining infections comprised respiratory infections (14.6%), urinary tract infections (8.0%), hospital-onset COVID-19 (6.8%), gastrointestinal infections other than CDI (4.7%), surgical site infections (3.2%), skin and soft tissue infections (2.8%), bloodstream or catheter-related infections (1.9%), and other infections (1.8%). The distribution is shown in Figure 4.

### 2.3. Comparison of CDI and Non-CDIs

**Figure 5:**
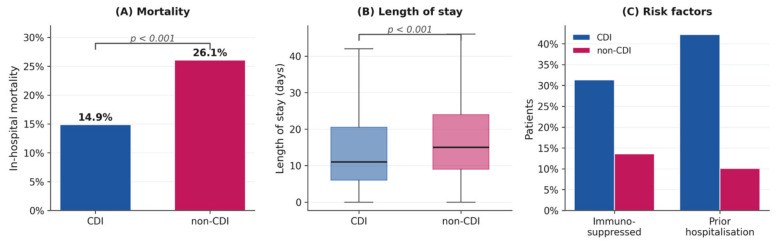
Clinical profile of CDI versus non-CDIs: (**A**) mortality, (**B**) length of stay, and (**C**) risk factors.

CDIs and non-CDIs differed markedly across the main clinical dimensions examined (Table 1, Figure 5). Patients with CDI were older than those with non-CDIs (median age 69 versus 64.5 years; Mann–Whitney U test, *p* < 0.001) and more frequently carried markers of prior healthcare contact, including immunosuppression (31.3% versus 13.6%) and prior hospitalisation within the preceding year (42.2% versus 10.1%; both *p* < 0.001). Despite this risk profile, in-hospital mortality was substantially lower in CDI than in non-CDIs (14.9% versus 26.1%; *p* < 0.001), and the median length of stay was shorter (11 versus 15 days; *p* < 0.001). The length of stay differed significantly across the infection groups overall (Kruskal–Wallis H = 149.8, degrees of freedom 8, *p* < 0.001). Dunn post hoc tests with Holm correction indicated that CDI differed from each non-CDI group except the heterogeneous “Other” category, whereas most pairwise comparisons among the non-CDI groups were not significant.

### 2.4. Antibiotic Exposure and Treatment

Antibiotic exposure during the current admission before symptom onset was strongly concentrated in CDI: 741 of 1620 *C. difficile* cases (45.7 percent) had documented prior antibiotic use, compared with 1 of 1258 non-CDI cases (0.1 percent; *p* < 0.001). Among the 713 CDI cases with a recorded treatment regimen, most received a single course of a single agent (251 cases) or multiple courses, either of a single agent (176) or of antibiotic combinations (167), with the remainder receiving a single course of combinations (119). A concurrent non-CDI was recorded in 127 CDI patients, and 7.0 percent of all notifications involved more than one infection type. These patterns identify antibiotic exposure as the principal modifiable antecedent of CDI in the cohort.

### 2.5. Determinants of In-Hospital Mortality

**Figure 6:**
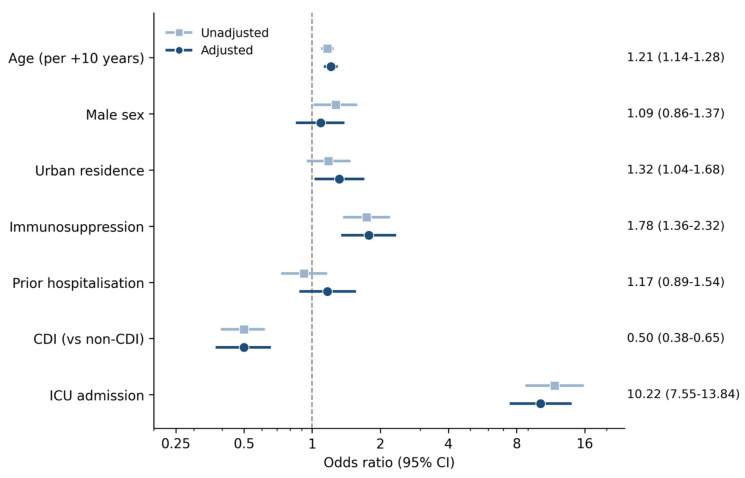
Unadjusted and adjusted odds ratios for in-hospital mortality (forest plot).

In the multivariable logistic regression model (2315 patients, 451 deaths), admission to an intensive care unit was the strongest predictor of in-hospital mortality (aOR 10.22, 95% CI 7.55–13.84), followed by older age (aOR 1.21 per additional 10 years, 95% CI 1.14–1.28), immunosuppression (aOR 1.78, 95% CI 1.36–2.32), and urban residence (aOR 1.32, 95% CI 1.04–1.68); male sex and prior hospitalisation were not independent predictors. CDI remained associated with substantially lower mortality than non-CDI after adjustment, including for ICU admission (aOR 0.50, 95% CI 0.38–0.65) (Table 2, Figure 6). ICU patients accounted for 305 cases (10.6 percent) and had markedly higher mortality than other wards (65.1 versus 13.7 percent). The model was globally significant (likelihood-ratio chi-square 390.3, degrees of freedom 7, *p* < 0.001), with a Nagelkerke R-squared of 0.247 and good discrimination (AUC 0.769, five-fold cross-validated 0.759), a substantial improvement over the model without ward type. Discharge status was missing for 562 patients (19.5 percent) and was more frequent for non-CDI than for CDIs (24.3 versus 15.9 percent; *p* < 0.001). The lower mortality associated with CDI was robust to the boundary assumptions that all missing outcomes were deaths (aOR 0.43, 95% CI 0.36–0.51) or survivals (aOR 0.40, 95% CI 0.32–0.50). The association reversed only under the most extreme and biologically implausible scenario, in which every missing CDI case was assumed to have died and every missing non-CDI case to have survived (aOR 1.36, 95% CI 1.12–1.65).

**Figure 7:**
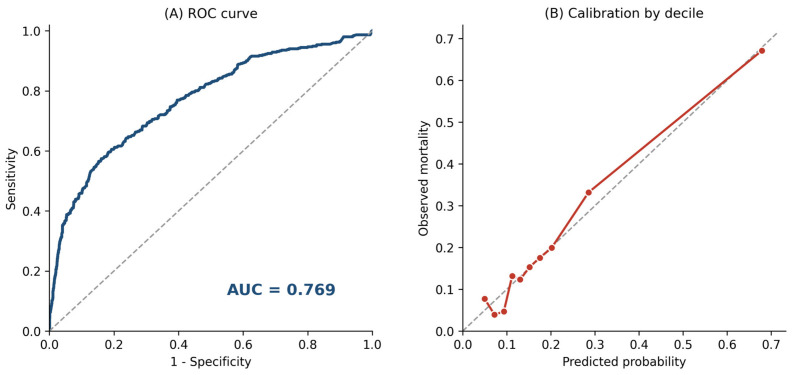
Discrimination and calibration of the multivariable mortality model. (**A**) ROC curve. (**B**) Calibration by decile.

**Figure 8:**
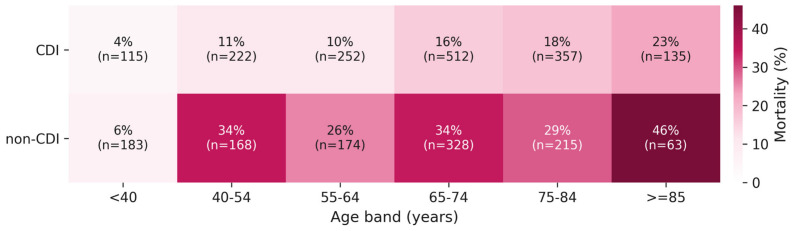
In-hospital mortality by age band and infection type.

Discrimination and calibration are shown in Figure 7: the receiver operating characteristic curve corresponded to an AUC of 0.769, and the decile calibration plot showed reasonable agreement between predicted and observed mortality, with a borderline Hosmer-Lemeshow test that is expected at this sample size and is best interpreted alongside the graphical calibration. All variance inflation factors were below 1.3, and hospital-clustered robust standard errors preserved the associations for age and CDI. Using the Levin formula, the population attributable risk for in-hospital mortality was 15.3 percent for immunosuppression. Stratifying mortality by age confirmed a consistent gradient in both groups, but non-CDIs carried higher mortality within every age band; the difference was most pronounced in middle-aged and older patients, where non-CDI mortality reached 34 to 46 percent against 16 to 23 percent for CDI (Figure 8).

### 2.6. Time-to-Event and Competing-Risks Analysis of Mortality

**Figure 9:**
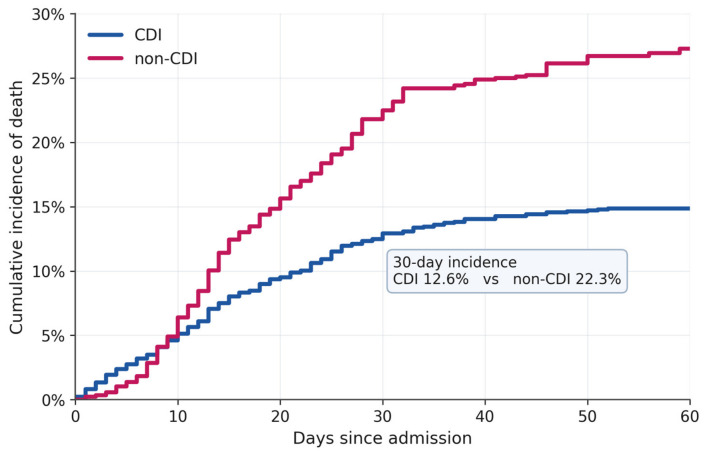
Cumulative incidence of in-hospital death by infection type (competing-risks analysis).

Kaplan–Meier analysis confirmed lower in-hospital mortality over time for CDI than for non-CDIs (log-rank *p* < 0.001). In the Cox proportional hazards model, the hazard of in-hospital death increased with age (HR 1.16, 95% CI 1.10–1.23) and prior hospitalisation (HR 1.37, 95% CI 1.09–1.71), was highest for ICU admission (HR 3.07, 95% CI 2.50–3.77), and was lower for CDI (HR 0.74, 95% CI 0.60–0.91), with the proportional hazards assumption satisfied for all covariates (Table 3). Because discharge alive competes with in-hospital death, a competing-risks analysis was also performed; the 30-day cumulative incidence of in-hospital death was 12.6 percent for CDI and 22.3 percent for non-CDIs (Figure 9), confirming the direction and magnitude of the difference while avoiding the overestimation inherent in censoring discharge.

**Figure 10:**
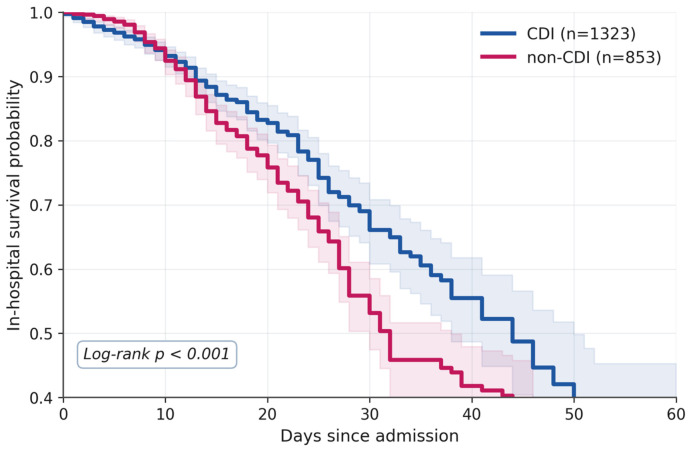
In-hospital survival by infection type (Kaplan–Meier curves).

The Kaplan–Meier curves separated within the first days of admission and remained distinct throughout the hospital stay, with consistently higher survival for CDI than for non-CDIs (Figure 10).

### 2.7. Length of Stay

Length of stay showed substantial overdispersion (Poisson dispersion 17.9) and was therefore modelled with negative binomial regression (Table 3). CDI was associated with a shorter stay than non-CDI (IRR 0.85, 95% CI 0.77–0.93), whereas male sex (IRR 1.20, 95% CI 1.10–1.31) and immunosuppression (IRR 1.16, 95% CI 1.04–1.28) were associated with a longer stay; ICU admission and age were not independently associated.

### 2.8. Hospital-Level Variation in Reported CDI Proportion

**Figure 11:**
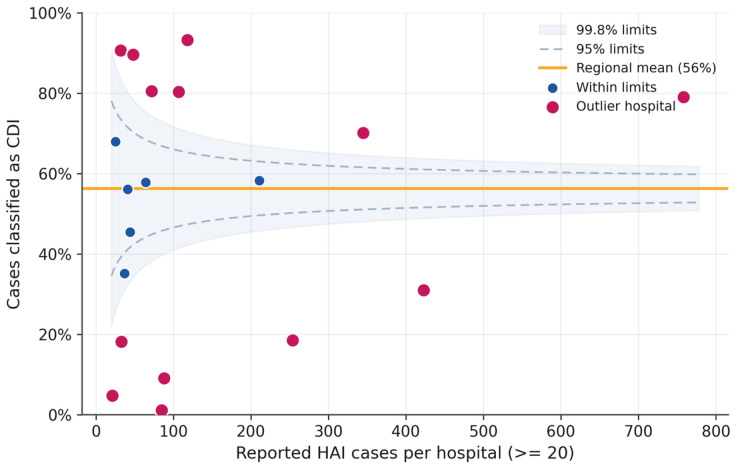
Hospital-level variation in the reported CDI proportion (funnel plot).

Thirty-two hospitals reported cases in 2024. Among institutions with at least 20 reported cases, the proportion of cases classified as CDI varied widely, from approximately 1 percent to 93 percent, against a regional mean of 56.3 percent. In the funnel plot, most hospitals fell outside the 99.8 percent control limits expected under random variation, indicating that the dominance of CDI was not uniform but driven by pronounced and systematic heterogeneity between institutions in the apparent capture of non-CDIs (Figure 11).

## 3. Discussion

### 3.1. Principal Findings

This patient-level, multicenter analysis of healthcare-associated infections in post-pandemic Southeast Romania produced four principal findings. First, the reported HAI profile was overwhelmingly dominated by *Clostridioides difficile* infection, which accounted for more than half of all cases. Second, CDIs and non-CDIs behaved as two distinct clinical phenotypes, differing in age, prior healthcare exposure, length of stay, and outcome. Third, in-hospital mortality was driven by intensive care admission, advancing age, and immunosuppression, and was paradoxically lower for CDI than for non-CDIs across crude, adjusted, time-to-event, and competing-risks analyses, including after adjustment for intensive care admission. Fourth, the reported proportion of CDI varied enormously between hospitals, far beyond what random variation could explain, pointing to heterogeneous surveillance performance rather than genuine epidemiological differences.

### 3.2. CDI Over-Representation and the Under-Reporting of Non-CDIs

The most striking finding was that CDI represented 56 percent of reported HAIs, against a proportion of approximately 10 percent in European point-prevalence surveys [7,8,10]. This discrepancy is most plausibly explained not by an exceptional regional burden of CDI but by the selective ascertainment of an infection that is laboratory-confirmable and subject to mandatory notification, while other HAIs depend on more complex, resource-intensive, and clinician-initiated detection [26,27], although the present design cannot formally exclude a genuinely higher local burden of CDI. This interpretation is consistent with the long-standing observation that apparently low or skewed HAI figures in Central and Eastern Europe reflect the limitations of surveillance rather than a smaller true burden [20,21]. The pattern observed here provides an empirical, patient-level demonstration of that phenomenon: the system reliably captures one non-traditional HAI while a large fraction of conventional device-associated and procedure-associated infections appear to remain undetected [26,30].

### 3.3. The Mortality Paradox

The clinical characteristics of CDI in this cohort, namely older patients with high frequencies of prior hospitalisation, immunosuppression, and antisecretory therapy, are consistent with the established risk profile of the infection [31]. Despite this, CDI carried a substantially lower risk of in-hospital death than non-CDIs, an association that persisted after adjustment (adjusted odds ratio 0.50) and in the competing-risks analysis (30-day cumulative incidence 12.6 versus 22.3 percent). Rather than implying that CDI is benign, this paradox indicates that the deadliest HAIs are precisely those that the surveillance system captures least completely, so that the true mortality burden is concentrated in the under-reported non-CDIs. The independent mortality determinants identified here, intensive care admission, advancing age, and immunosuppression, are congruent with the broader literature on host susceptibility, in which immune dysfunction, ageing, and chronic comorbidity strongly modulate the outcome of infection [32,33,34]. Part of the higher mortality of non-CDIs reflected their concentration in intensive care; after adjustment for ICU admission, CDI nevertheless remained independently associated with lower in-hospital mortality, so the paradox is attenuated but not explained away by case mix.

### 3.4. Heterogeneity of Surveillance Quality Across Hospitals

The funnel plot showed that the dominance of CDI was not a uniform regional feature but the product of pronounced between-hospital variation, with most institutions lying outside the 99.8 percent control limits [35]. Such variation is unlikely to reflect true differences in infection epidemiology and more probably indicates differences in diagnostic capacity, reporting culture, and the maturity of local infection-control structures, factors known to shape institution-level HAI indicators [36,37]. Comparable under-ascertainment of *C. difficile* has been documented across Europe, where wide variation in testing and reporting practice produces large differences in apparent CDI rates and an estimated tens of thousands of undiagnosed cases each year [22,23,24]. Treating the reported CDI proportion as a surveillance-quality indicator, therefore, offers a simple and scalable way to flag institutions where non-CDIs are likely to be under-detected.

### 3.5. Implications for Practice and Policy

The concentration of prior antibiotic exposure in CDI, present in nearly half of CDI cases and virtually absent in non-CDIs, is consistent with the central role of antibiotic pressure in the pathogenesis of CDI and underlines antimicrobial stewardship as the principal preventive lever. Because CDI is the most completely captured HAI, while the more lethal non-CDIs are under-detected, stewardship and improved ascertainment are complementary rather than competing priorities.

These findings carry direct implications for infection prevention and control. They argue for strengthening the ascertainment of non-CDIs through standardised case definitions, dedicated surveillance personnel, and laboratory capacity, so that the reported profile better reflects the true distribution of HAIs [1,7,20]. Institution-level benchmarking, using indicators such as the CDI-to-total ratio together with funnel-plot control limits, could identify hospitals in need of targeted support [4]. More broadly, the identification of the determinants of adverse outcomes is a prerequisite for designing targeted preventive interventions, an approach that has proved valuable across many areas of clinical practice [38]. Because under-detected non-CDIs carry the greater mortality and contribute disproportionately to antimicrobial resistance, improving their capture is not merely an administrative goal but a patient-safety and stewardship priority [4,5].

### 3.6. Strengths and Limitations

The study has several strengths, including its multicentre regional scope, its patient-level granularity, and the use of robust analytical methods, in particular a competing-risks framework appropriate to in-hospital outcomes [39]. Several limitations should be acknowledged. First, the inference of under-reporting is interpretive rather than directly demonstrated. The dataset comprised notified cases without a denominator of admissions or patient-days, so incidence could not be estimated, and the proportion of cases classified as CDI is a compositional measure: a high value is consistent with under-detection of other HAIs but does not prove it, and a genuinely higher local burden of CDI or differences in case mix cannot be fully excluded. For the same reason, the contrast between the observed 56 percent and the European figure of approximately 10 percent is indicative rather than exact, since the latter derives from point-prevalence surveys with a different design and denominator. Second, discharge status was missing for about one-fifth of patients and was more frequent among non-CDIs, so the missingness was not random; although the direction of the lower mortality associated with CDI was robust to plausible boundary assumptions, it was sensitive to extreme differential misclassification, and the magnitude of the association should therefore be interpreted with caution. Third, the dataset lacked microbiological detail, such as organism identification, resistance phenotype, and CDI ribotype, and did not capture patient-reported outcomes or quality of life [40,41]. Fourth, residual confounding from unmeasured comorbidity and disease severity cannot be excluded, although the inclusion of ward type improved the explanatory performance of the mortality model. Finally, the 2025 data were incomplete and were therefore not analysed. Concurrent non-CDI was recorded in 127 CDI patients, but the surveillance dataset did not capture organism identification, resistance phenotype, or ribotype, so the contribution of specific pathogens and of resistance could not be assessed.

### 3.7. Future Directions

Two avenues follow naturally from this work. The first is longitudinal: extending the analysis across the full 2020 to 2024 period would allow the post-pandemic landscape to be compared with pandemic and pre-pandemic patterns and would permit a formal assessment of seasonality and of the lasting effect of COVID-19 on HAI detection and reporting [12,13,16,18,25]. The second is methodological: the limitations of manual, selective ascertainment demonstrated here strengthen the case for automated and increasingly data-driven surveillance, including electronic case detection and the application of artificial intelligence and machine learning to clinical data [28,29,42,43]. Such approaches are already being explored for risk prediction, screening, and predictive analytics across diverse clinical domains [44,45,46,47,48,49], and rest on broader advances in predictive and optimisation modelling [50]; applied to infection surveillance, they could reduce the dependence on infection type for case capture and yield a more complete and less biassed picture of the true burden of healthcare-associated infections.

## 4. Materials and Methods

### 4.1. Study Design and Setting

A retrospective, observational, cross-sectional analysis of individual patient-level healthcare-associated infection (HAI) surveillance records from a multicentre regional network in the South-East region of Romania was conducted. The network comprises reporting hospitals located in five counties (Constanța, Galați, Tulcea, Buzău, and Brăila), spanning tertiary county emergency hospitals, municipal and specialty hospitals, and chronic-care and rehabilitation units. The study was designed and reported in accordance with the Strengthening the Reporting of Observational Studies in Epidemiology (STROBE) statement for cross-sectional studies [51].

### 4.2. Data Source and Study Period

Data were obtained from the regional HAI surveillance database, in which each record corresponds to a notified case and contains demographic, clinical, microbiological, exposure, and outcome information. Although the database covered the period from 2020 to 2025, the present analysis focused on the complete 2024 reporting year, which provided a full and stable post-pandemic cohort. Records for 2025 were incomplete at the time of extraction and were considered to be undergoing ongoing update; they were therefore excluded from the primary analysis and reserved for a separate longitudinal study.

### 4.3. Case Definitions and Infection Classification

Cases were identified and classified according to the standardised case definitions used in the national surveillance methodology, which are harmonised with the European criteria for HAI surveillance [7,20]. Each case was assigned to a single principal infection type. For analytical purposes, infections were grouped into *Clostridioides difficile* infection (CDI) and non-CDI HAIs. CDI was defined on the basis of compatible clinical presentation together with laboratory confirmation (detection of toxin A and/or B, nucleic acid amplification for toxin genes, or characteristic endoscopic or histopathological findings) and is subject to mandatory notification [27]. Non-CDI HAIs were further categorised as respiratory, urinary, surgical site, bloodstream or catheter-related, gastrointestinal other than CDI, skin and soft tissue, and other infections. Hospital-onset COVID-19 was retained as a distinct category to characterise the residual pandemic contribution to the reported profile.

### 4.4. Variables and Outcomes

Demographic variables comprised age, sex, and area of residence (urban or rural). Exposure and host variables included immunosuppression, hospitalisation within the preceding year, antisecretory therapy in the previous three months, mechanical ventilation, the presence of severe chronic comorbidity, ward type (admission to an intensive care unit versus other wards), and antibiotic exposure during the current admission before symptom onset. Recent antisecretory therapy was available as a CDI-specific surveillance variable and was therefore summarised descriptively for CDI cases but not treated as a comparable exposure across CDI and non-CDI. The principal exposure of interest for comparative analyses was infection type (CDI versus non-CDI). The primary outcome was in-hospital mortality, derived from the recorded discharge status, with death classified against all other discharge dispositions (improved, cured, stable, transferred, or worsened). Secondary outcomes were length of hospital stay, expressed in days, and time from admission to in-hospital death.

### 4.5. Data Cleaning

Prior to analysis, the dataset was screened for implausible values. Recorded ages outside the range of 0 to 110 years and lengths of stay outside the range of 0 to 365 days were treated as missing. Categorical exposure fields were standardised to consistent binary coding, and free-text and placeholder entries denoting absent information were harmonised to a single missing-value indicator. No imputation was applied for the primary analysis, and the number of records contributing to each model is reported alongside the corresponding estimates.

### 4.6. Statistical Analysis

Continuous variables were summarised as the mean with standard deviation or as the median with interquartile range (IQR), according to their distribution, and categorical variables as absolute frequencies and percentages. The distribution of continuous variables was assessed with the Shapiro–Wilk test, which indicated non-normality and supported the use of non-parametric methods. Between-group comparisons used the chi-square test or Fisher’s exact test for categorical variables, the Mann–Whitney U test for two-group comparisons of continuous variables, and the Kruskal–Wallis test, with Dunn post hoc comparisons and Holm correction across more than two infection groups. Monotonic associations between continuous variables were examined with the Spearman rank correlation coefficient.

Determinants of in-hospital mortality were examined using univariable and multivariable binary logistic regression, with results expressed as unadjusted and adjusted odds ratios (OR and aOR) with 95% confidence intervals (CI). The overall significance of the multivariable model was assessed with the omnibus likelihood-ratio chi-square test, explained variation with the Nagelkerke R-squared, and goodness of fit with the Hosmer-Lemeshow test [52]. Discrimination was quantified by the area under the receiver operating characteristic curve (AUC), with a 95% CI obtained from 2000 bootstrap resamples and internal validity assessed by five-fold stratified cross-validation; calibration was evaluated graphically across deciles of predicted risk. A patient-level risk score was defined as the predicted probability of in-hospital death from the multivariable logistic model, and the receiver operating characteristic curve was constructed across all probability thresholds; the AUC was reported as a global measure of discrimination, and no single clinical cut-off was applied, since the model was intended to be explanatory rather than a diagnostic classifier. Multicollinearity was assessed using variance inflation factors, and hospital-clustered robust standard errors were computed as a sensitivity analysis. The population attributable risk for the principal modifiable determinants was estimated using the Levin formula [53].

The influence of missing discharge status on the mortality analysis was examined by comparing the pattern of missingness between CDIs and non-CDIs and by refitting the multivariable model under boundary and extreme-case assumptions, in which the missing outcomes were assigned first uniformly and then differentially between the two groups.

Time-to-event analyses of in-hospital death used the Kaplan–Meier estimator with the log-rank test for unadjusted comparisons and the Cox proportional hazards model for adjusted hazard ratios (HR), with the proportional hazards assumption verified using scaled Schoenfeld residuals. Because discharge alive precludes the observation of in-hospital death, a competing-risks framework was additionally applied, estimating the cumulative incidence of death with the Aalen-Johansen estimator and treating discharge alive as a competing event, in line with current recommendations for the analysis of hospital-acquired infection data [39,54]. The length of stay was modelled with negative binomial regression, selected over Poisson regression on the basis of substantial overdispersion, with effects reported as incidence rate ratios (IRR) with 95% CI. Between-hospital variation in the reported CDI proportion was evaluated with a funnel plot constructed according to the method of Spiegelhalter, using 95 percent and 99.8 percent control limits to identify institutions lying beyond the bounds expected from random variation [35].

Statistical analyses were carried out in Python (version 3.12) using established open-source libraries for data management, regression modelling, survival analysis, and competing-risks estimation. A two-sided p-value below 0.05 was considered statistically significant.

### 4.7. Ethical Considerations

This study used routinely collected, de-identified case-based surveillance data obtained as part of national communicable disease surveillance activities. The analysis did not involve any intervention, contact with patients, or access to directly identifying information. Data were processed in accordance with the General Data Protection Regulation (EU 2016/679) and with the applicable Romanian legal framework for public-health surveillance. Under Romanian legislation, ethical approval and individual informed consent are not required for secondary analyses of anonymised surveillance data collected for statutory public-health purposes. The study was conducted in accordance with the principles of the Declaration of Helsinki.

## 5. Conclusions

In this patient-level, multicentre analysis of post-pandemic healthcare-associated infection surveillance in Southeast Romania, the reported infection profile was dominated by *Clostridioides difficile* infection, which accounted for more than half of all cases, far above the proportion expected from European surveillance. This pattern is best understood not as an exceptional regional burden of CDI but as a signature of selective ascertainment, in which an easily confirmable and mandatorily notified infection is captured far more completely than the conventional device-associated and procedure-associated infections that depend on more complex detection.

The clinical consequences of this bias are not neutral. In-hospital mortality was independently driven by intensive care admission, advancing age, and immunosuppression, and was consistently lower for CDI than for non-CDIs across adjusted, time-to-event, and competing-risks analyses. The deadliest infections were therefore precisely those least completely captured, meaning that the true mortality burden of healthcare-associated infections is concentrated where the surveillance system is least able to see it. The marked between-hospital variation in the reported CDI proportion further indicated that this under-detection is heterogeneous, and that the CDI-to-total ratio can serve as a simple indicator of surveillance quality.

Taken together, these findings reframe persistently low or skewed national HAI figures as a measurement problem rather than evidence of a smaller burden. Strengthening the ascertainment of non-CDIs, through standardised case definitions, dedicated personnel, laboratory capacity, institution-level benchmarking, and a progressive shift toward automated and data-driven surveillance, should be regarded as a strategic patient-safety and antimicrobial-stewardship priority, essential to building a more accurate and more equitable picture of healthcare-associated infections in Romania.

## Figures and Tables

**Table 1:** Baseline characteristics of the 2024 cohort, overall and by infection group (CDI versus non-CDI).

Characteristic	Overall (N = 2878)	CDI (n = 1621)	Non-CDI (n = 1257)	*p*
Age, years, median (IQR)	68 (52-76)	69 (57-78)	64 (42-75)	<0.001
Age >= 65 years, n (%)	1673 (58.2)	1045 (64.5)	628 (50.0)	<0.001
Male sex, n (%)	1517 (52.9)	842 (52.1)	675 (54.0)	0.334
Urban residence, n (%)	1651 (57.6)	1007 (62.3)	644 (51.6)	<0.001
ICU admission, n (%)	305 (10.6)	83 (5.1)	222 (17.6)	<0.001
Length of stay, days, median (IQR)	13 (7–22)	11 (6–20)	15 (9–24)	<0.001
Immunosuppression, n (%)	679 (23.6)	508 (31.3)	171 (13.6)	<0.001
Prior hospitalisation < 1 y, n (%)	811 (28.2)	684 (42.2)	127 (10.1)	<0.001
In-hospital death, n (%)	451 (19.5)	203 (14.9)	248 (26.1)	<0.001

**Table 2:** Univariable and multivariable logistic regression for in-hospital mortality.

Predictor	Unadjusted OR (95% CI)	*p*	Adjusted OR (95% CI)	*p*
Age (per +10 years)	1.17 (1.11–1.23)	<0.001	1.21 (1.14–1.28)	<0.001
Male sex	1.27 (1.03–1.56)	0.026	1.09 (0.86–1.37)	0.489
Urban residence	1.18 (0.96–1.46)	0.124	1.32 (1.04–1.68)	0.023
Immunosuppression	1.74 (1.39–2.18)	<0.001	1.78 (1.36–2.32)	<0.001
Prior hospitalisation	0.92 (0.74–1.15)	0.479	1.17 (0.89–1.54)	0.265
CDI (vs. non-CDI)	0.50 (0.40–0.61)	<0.001	0.50 (0.38–0.65)	<0.001
ICU admission	11.79 (8.88–15.67)	<0.001	10.22 (7.55–13.84)	<0.001

**Table 3:** Secondary-outcome models: time to in-hospital death (Cox proportional hazards) and length of stay (negative binomial).

Predictor	Cox HR, Death (95% CI)	*p*	NB IRR, Length of Stay (95% CI)	*p*
Age (per +10 years)	1.16 (1.10–1.23)	<0.001	1.01 (0.99–1.03)	0.496
Male sex	0.92 (0.76–1.11)	0.368	1.20 (1.10–1.31)	<0.001
Immunosuppression	1.20 (0.97–1.48)	0.090	1.16 (1.04–1.28)	0.006
Prior hospitalisation	1.37 (1.09–1.71)	0.006	not in model	-
CDI (vs. non-CDI)	0.74 (0.60–0.91)	0.005	0.85 (0.77–0.93)	<0.001
ICU admission	3.07 (2.50–3.77)	<0.001	1.13 (0.99–1.30)	0.076

## Data Availability

The data presented in this study are not publicly available, as they were obtained as part of national communicable disease surveillance and provided by the National Institute of Public Health (INSP-CNSCBT) upon official request, for use with attribution to the source of provenance.

## Abbreviations

The following abbreviations are used in this manuscript:

aOR	Adjusted odds ratio
AUC	Area under the curve
CDI	*Clostridioides difficile* infection
CI	Confidence interval
COVID-19	Coronavirus disease 2019
df	Degrees of freedom
ECDC	European Centre for Disease Prevention and Control
HAI	Healthcare-associated infection
HR	Hazard ratio
IQR	Interquartile range
IRR	Incidence rate ratio
NB	Negative binomial
OR	Odds ratio
SD	Standard deviation
STROBE	Strengthening the Reporting of Observational Studies in Epidemiology
WHO	World Health Organization
